# The Impact of Bariatric-Surgery-Induced Weight Loss on Patients Undergoing Liver Transplant: A Focus on Metabolism, Pathophysiological Changes, and Outcome in Obese Patients Suffering NAFLD-Related Cirrhosis

**DOI:** 10.3390/jcm11185293

**Published:** 2022-09-08

**Authors:** Gerardo Sarno, Luigi Schiavo, Pietro Calabrese, Ludwig Álvarez Córdova, Evelyn Frias-Toral, Gabriela Cucalón, Eloisa Garcia-Velasquez, Vanessa Fuchs-Tarlovsky, Vincenzo Pilone

**Affiliations:** 1 San Giovanni di Dio e Ruggi D’Aragona University Hospital, Scuola Medica Salernitana, 84131 Salerno, Italy; 2 Center of Excellence of Bariatric Surgery of the Italian Society of Obesity Surgery and Metabolic Disease (SICOB), Unit of General and Emergency Surgery, University Hospital San Giovanni di Dio e Ruggi d’Aragona, P.O. Gaetano Fucito Mercato San Severino, 84085 Salerno, Italy; 3 Department of Medicine, Surgery and Dentistry, Scuola Medica Salernitana, University of Salerno, 84081 Baronissi, Italy; 4 Carrera de Nutrición y Dietética, Facultad de Ciencias Médicas, Universidad Católica De Santiago de Guayaquil, Av. Pdte. Carlos Julio Arosemena Tola, Guayaquil 090615, Ecuador; 5 School of Medicine, Universidad Católica Santiago de Guayaquil, Av. Pdte. Carlos Julio Arosemena Tola, Guayaquil 090615, Ecuador; 6 Universidad de Especialidades Espìritu Santo, Samborondòn 0901952, Ecuador; 7 Lifescience Faculty, ESPOL Polytechnic University, Escuela Superior Politécnica del Litoral (ESPOL), Campus Gustavo Galindo Km. 30.5 Vía Perimetral, Guayaquil 090615, Ecuador; 8 Clinical Nutrition Service, Grupo Hospitalario Kennedy, Guayaquil 090615, Ecuador; 9 Clinical Nutrition Service, Hospital General de Mexico, Ciudad de México 06720, Mexico

**Keywords:** obesity, bariatric surgery, NAFLD, cirrhosis, liver transplantation

## Abstract

Because of their condition, patients with morbid obesity develop several histopathological changes in the liver, such as non-alcoholic fatty liver disease (NAFLD), non-alcoholic steatohepatitis (NASH), cirrhosis, and end-stage liver disease (ESLD). Hence, a liver transplant (LT) becomes an opportune solution for them. Due to many challenges during the perioperative and postoperative periods, these patients are recommended to lose weight before the surgery. There are many proposals to achieve this goal, such as intragastric balloons and many different bariatric surgery (BS) procedures in combination with a preparation diet (very-low-calorie diet, ketogenic diet, etc.). All the interventions focus on losing weight and keeping the continuity and functionality of the digestive tract to avoid postoperative complications. Thus, this review analyzes recent publications regarding the metabolic and pathophysiological impacts of BS in LT patients suffering from NAFLD-related cirrhosis, the effect of weight loss on postoperative complications, and exposes the cost-effectiveness of performing BS before, after, and at liver transplantation. Finally, the authors recommend BS before the LT since there are many positive effects and better outcomes for patients who lose weight before the procedure. Nevertheless, further multicentric studies are needed to determine the generalizability of these recommendations due to their impact on public health.

## 1. Introduction

Malnutrition due to excess food consumption has been associated with many adverse events for overall health related to insulin resistance (IR), dyslipidemia, and hypertension. Non-alcoholic fatty liver disease (NAFLD) is the most common condition of metabolic syndrome (MetS) and is intimately related to the prolonged intrahepatic inflammation linked to adiposity and IR [1,2].

NAFLD is a frequent condition due to the obesity epidemic. NAFLD has been defined as fatty infiltration of the liver exceeding 5% in histology in the absence of previous or ongoing alcohol consumption and/or drug and/or virus infection [1]. Additionally, it includes a spectrum of histopathological alterations going from steatosis to non-alcoholic steatohepatitis (NASH), followed by cirrhosis with end-stage liver disease (ESLD) [1]. Most patients with NAFLD are at a high risk of developing chronic liver disease complications. In patients with fibrotic NASH or cirrhosis, approximately 40% of these patients will die within the next 15 years from any cause related to liver-related complications [1]. The chronic inflammation associated with obesity due to excessive intra-abdominal fat tissue (IFT) leads to increased production of adipokines (IL-6, TNFa, monocyte chemoattracting protein-1, and plasminogen activator inhibitor-1) with proinflammatory, pro-fibrogenic, pro-angiogenic, and pro-oxidant effects on most tissues [1].

It is noteworthy that obesity-related liver complications such as NAFLD, NASH, or associated hepatocellular carcinoma have been prevalent in countries such as the US [3,4]. It has been stated that the severity of IR and the histological grade of steatosis appeared to be the factors more tightly associated with the liver disease progression [2]. However, weight loss has been shown to benefit patients with advanced chronic liver disease.

Bariatric surgery (BS) is considered a corrective option for morbid obesity and metabolic complications, with good results in the long term [5]. Additionally, is advised for surgical management of NALFD patients. BS can enhance liver histology and fibrosis [6]. The European Association for the Study of the Liver guidelines especially recommend it when lifestyle changes and medical treatment are unsuccessful [7]. The American Association for the Study of Liver Diseases recommended foregut bariatric procedures in obese individuals with NAFLD and NASH [8].

Patients with morbid obesity listed for BS commonly present an expanded steatotic liver, making the procedure challenging for the surgeon. Liver segments II and III and the type and location of the intraabdominal fat make this procedure more complicated. The storage of free fatty acid in the type of triglycerides is a frequent condition in NAFLD in obese individuals [9]. Longer operative time, intra-operative bleeding, anastomotic leakage, and not appropriate bariatric anatomy due to an increased IFT are some of the challenges for the bariatric surgeon because of a decreased work capacity that may complicate the surgical task. Consequently, preoperative nutritional interventions to reduce body weight and hepatomegaly can benefit the surgery procedure and patient evolution [10].

The most feared complications of BS are severe malnutrition and liver failure because they are considered life-threatening sequence complications. In addition, it has been associated with a long intestinal bypass, biliopancreatic diversion, and protein deficiency leading to liver failure [3,11].

Liver transplantation (LT) is a surgical option for NALFD patients, especially in Western countries [12]. The frequency of obesity, sarcopenia, chronic kidney disease, and cardiovascular disease in patients with NASH predisposes to a high prevalence of post-transplant complications and increased graft loss [13]. The frequency of LT has increased to up to 33% of procedures in the last decade [14]. The main contraindication for this technique is morbid obesity, described by a body mass index (BMI) above 40 kg/m^2^. There is no agreement on cut-off points of the BMI index to recognize patients with a risk of complications. There is a debate about using BS to increase the chances for LT candidacy for individuals with morbid obesity [15].

Candidates for LT are requested to lose weight before being registered for the procedure. Very-low-calorie diets (VLCD) produce a catabolic state with a critical nutritional risk, and weight loss should be attached to the loss of fat-free mass (FFM). The quality of the diet and the quantity of protein is more important than the amount of Kcal to prevent muscle depletion. After BS, the energy offered is restricted, similar to the VLCD model. Therapeutic nutritional supervision should be safe and effective in reducing BMI, enhancing candidacy for LT, and improving liver injury [16]. The condition of sarcopenia and malnutrition can be related to hypermetabolism, advanced starvation state, and change in amino acid profile in cirrhosis patients [17]. One of the recent approaches for patients undergoing BS is the ketogenic diet which may reduce body weight, IFT, left hepatic lobe volume, and micronutrient deficiency [18]. However, this issue is still one of the common deficiencies that can complicate the surgical technique and increase morbidity due to postoperative complications [19]. This fact emphasizes the importance of a multidisciplinary team with collaborative nutritional assistance [20].

This review aims to present updated literature about the metabolic and pathophysiological effects of BS in LT recipients suffering from NAFLD-related cirrhosis. It analyzes the correlation between weight-loss intra- and postoperative complications and the outcomes of these procedures. Finally, it considers the cost-effectiveness of BS in LT.

## 2. Metabolic Changes and Liver Pathophysiology Related to Bariatric Surgery

BS is one obesity treatment that will help to maintain weight loss and comorbidity control. Initially, these operations aimed to achieve those goal; however, nowadays, it is well known that they have other advantages, especially in generating better metabolic control, organ physiology, and behavior changes. Currently, BS is no longer considered a restrictive or malabsorptive procedure but a metabolic procedure that induces complex physiological changes and gut adaptation that influence signaling pathways in several organs, including the liver and brain. These procedures help regulate satiety, hunger, body weight, glucose metabolism, and immune functions [21].

Obesity is a risk factor for developing NAFLD and NASH worldwide [22]. Both conditions are related to IR and dyslipidemia, which may cause MetS, and NAFLD is associated with chronic liver disease [23]. Losing weight is the most effective treatment for NAFLD. It is documented that almost a 3.5% weight loss is associated with a reduction in steatosis, and a 10% weight loss may even reverse necro-inflammation of the liver [21].

Energy restriction and a low carbohydrate diet help to improve IR, hepatic insulin sensitivity, glucose tolerance, inflammation, hepatic lipogenesis, oxidative stress, and even liver fibrosis [24,25]. It has been proved that BS, as one example of a low-calorie, low-carbohydrate diet, can help solve up to 85% of NASH and improve NAFLD. It enhances liver enzymes such as alkaline phosphatase, alanine, and aspartate transaminases, as well as γ-glutamyltransferase profiles in plasma and histological markers such as steatosis and fibrosis [26,27,28]. In a five-year follow-up with 101 patients with obesity who underwent BS and liver biopsies, the authors reported that 41.6% were diagnosed with NASH, and 47.8% had liver fibrosis. The weight loss percentage was 7.6% in the first year, 8.3% three years after, and 5% five years after the surgery. They found that both advanced and significant cirrhosis improved significantly [29]. In another study, Nickel et al. evaluated a group of 100 patients scheduled for laparoscopic SG or RYGB. They evaluated NAFLD using elastography and laboratory-based fibrosis scores. They found significant improvements in anthropometric parameters such as BMI, % of total weight loss, and the Edmonton obesity staging system. Liver stiffness improved significantly when comparing pre- and post-surgery in approximately 12.5 post-surgery (12.9 ± 10.4 vs. 7.1 kPa, *p* < 0.001). They found no association between weight loss and NAFLD, but they concluded that BS was a potentially successful treatment for NAFLD [30].

In one study that analyzed 150 biopsies obtained from patients who will undergo BS, there was a significant improvement in 53 of the follow-up biopsies obtained 192 days after surgery. The results showed that BS improves NAFLD significantly even though the liver histologic recovery was not significantly different according to the type of surgery (SG or RYGB) [31]. Another exciting result demonstrated with metabolic surgery is the effect that it may have on the gut microbiota and bile acids. An increase in bile acid concentrations was shown. Still, as the patient loses weight, the concentration levels appear to be lower, and it positively affects the liver, reflecting increasing VLDL levels and reducing triglycerides accumulation [21].

There is a systematic review about a rare but potentially deadly complication after BS that may lead to LF with the need for an LT. The authors described 14 studies, including 36 patients who underwent BS and had severe complications leading to LF; 32 had an LT, while 4 died while on the waiting list for LT. They concluded that this liver failure is infrequent but may need an LT, which may help restore liver function even if BS is reversed simultaneously [3]. A recent case was reported in which the patient developed severe malnutrition, bacterial overgrowth, and LF after one anastomosis gastric bypass undertaken following weight regain posterior to a laparoscopic SG. This case highlighted the relevance of quickly solving this patient’s severe malnutrition using an RYGB as a restrictive surgical option that does not lead to LF [11,32].

Those BSs performed when a patient already has a liver disease rarely worsen the liver condition, the need for an LT, and the progression of cirrhosis. BS has become helpful in patients with NAFLD. However, we should consider some reports about the intensification of liver disease after BS. The evidence indicates that a percentage of BS patients, especially those who will be submitted to a restrictive surgery, may experience a rapid affection on their liver function, mainly if their liver was already affected before surgery which may lead to the need for an LT [33]. The American Gastroenterological Association suggested that BS should be used when the LF is compensated but should be carefully considered in patients with potential extrahepatic comorbidities, including portal hypertension [34].

A recent meta-analysis regarding LT before and after BS concluded that LT is still considered a high-risk surgery associated with morbidity and mortality [35]. A few studies found in the literature showed that all LT post-bariatric surgery patients lost weight as expected. Some complications related to surgery, such as bile leakage or bleeding, and stable graft functions were observed. The mortality rate seems higher when the LT occurs after BS, but the immunosuppression protocol was unaffected because of the BS procedure [36,37].

**Figure 1:**
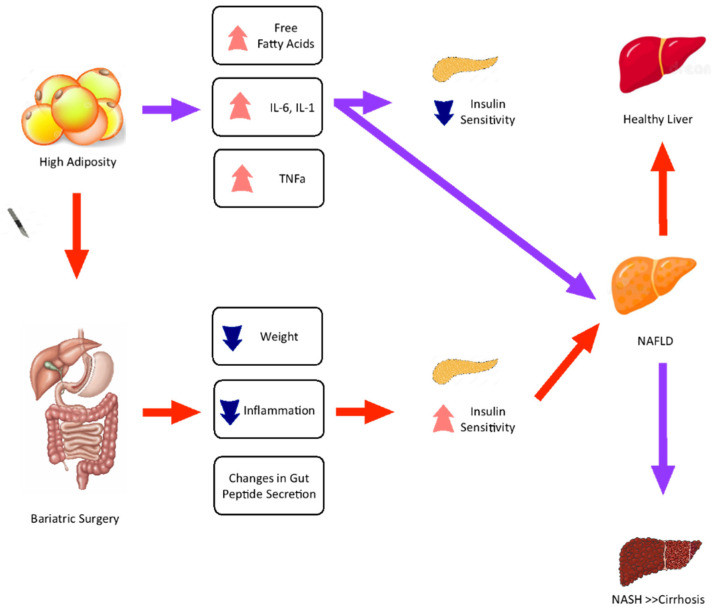
Mechanisms of protection and development of non-alcoholic fatty liver disease (NAFLD) in people living with obesity.

Figure 1 graphically explains that people living with obesity have more significant amounts of IFT that secretes proinflammatory cytokines such as IL-1 and IL-6, Tumor Necrosis Factor-alpha (TNFa), and free fatty acids (FFAs). This state may reduce insulin sensitivity and thus increase glycemia. This state of IR, along with other dietary factors, could compromise the ability of the liver to handle glucose and favor fat deposition in the liver.

In Figure 1, obesity and the typical accumulation of fat in adipose tissue, secretion of pro-inflammatory cytokines such as interleukins 1 and 6 (IL-1 and IL-6), as well as tumor necrosis factor-alpha (TNFa) and free fatty acids (FFAs), are shown by the orange arrows. As a consequence, insulin resistance increases along with glycemia. This metabolic state could reduce the ability of the liver to handle glucose and cause a predisposition to a higher synthesis of fatty acids that will deposit in the liver, thus causing NAFLD. A high-fructose diet has been reported to further increase fatty acid synthesis in the liver, thus, worsening NAFLD. NAFLD may progress into NASH, which causes hepatocyte damage and non-reversible cirrhosis. Bariatric surgery (green arrows) can reverse the evolution of NAFLD into NASH by improving insulin sensitivity with the consequent reduction of glucose influx into the liver and reducing fatty acid synthesis in this organ.

The inflammatory status increases oxidative stress, and consequently it damages the hepatic tissue. In some cases, the liver may become so damaged that it evolves into NASH. Consequently, NASH can progress into fibrosis and eventually to irreversible liver cirrhosis. BS can reverse the evolution of NAFLD into NASH (if there is no fibrosis or cirrhosis). One of the main mechanisms linked to this process is the improvement in insulin sensitivity from (1) a reduction in inflammation by reducing the amount of adipose tissue and (2) changes in gut peptide secretion that can improve satiety and decrease IR. These changes reduce the influx of glucose into the liver, allowing the hepatic tissue to handle the deposited fat [21].

## 3. Impact of Bariatric Surgery on Absorption and Effectiveness of Immunosuppressants

BS impacts the digestive tube anatomy directly, therefore, it is expected to cause changes related to the absorption of certain nutrients and drugs that we should consider to address the patient’s needs in this new condition. We must view that weight loss is related to improving comorbidities due to obesity; therefore, surgery is recommended in some cases. However, some techniques reduce intestinal absorptive surfaces such as those bypassing different parts of the intestine. The anatomic and physiological alterations of the gastrointestinal tract after BS may limit the oral bioavailability of various drugs [38,39,40].

Bypass surgery reduces the absorption surface of the intestine, but that does not necessarily mean that it will affect absorption. Thus, it may affect it because part of the bypassed intestine is where some of the metabolizing enzymes are. As a result, the absorption will be affected. After being absorbed, the drugs will be subject to intestinal and hepatic metabolism, where the cytochrome p450 (CYP) metabolizes most medicines [38,41].

In a recent review, Miedziaszczyk et al. [41] described the effect of BS on drug, vitamin, and mineral absorption and stated that the anatomy changes that start with this type of surgery are expected to affect absorption. After reviewing the current publications, the authors concluded that in BS there is a decrease in the time to maximum plasma concentration of the different micronutrients (tmax) and a reduction in maximum plasma concentration levels of the micronutrients (Cmax) compared to the levels observed in controls. They also stated that vitamin and mineral deficiencies were present in the studied population. These drugs’ effects vary depending on the drug properties and type of surgical procedure [41]. Chen et al. [42] presented the available literature about drug pharmacokinetics after BS in another review. They suggested that there are no specific papers that demonstrate the effect of the surgery on each drug. Hence, clinicians should be aware of monitoring postoperative changes in gastrointestinal physiology and weight loss. These changes may reduce, increase, or not alter the effects on drug exposure levels. Clinicians should monitor drug efficacy and safety by employing biomarkers to avoid adverse events [42].

BS can be beneficial in patients that need LT because of the deleterious consequences of obesity; BS seems to be more useful before transplantation. If a patient loses weight before LT, he will have more benefits in terms of survival and quality of life and fewer complications [43]. It is essential to consider the risks of taking medication after BS and the need for organ transplantation; the interaction that may occur with immunosuppressant drugs remains unclear. Chenhsu et al. compared cyclosporin levels in patients after LT who underwent bariatric surgery 26 years before the transplant. They found that the drug blood levels were 50% lower in the BS patients than in the healthy controls. Therefore, their recommendation is to consider prescribing a higher cyclosporin dose in patients who will undergo BS to achieve the expected effect [44].

In another study, Rogers et al. [45] evaluated the pharmacokinetics of immunosuppressant drugs in patients submitted to BS. They registered that those who were transplanted maintained their current medication plan. The Cmax, Tmax, and the area under the attention of the drug in plasma as opposed to the time curve (AUC 0–12 and AUC 0–infinite) met the standards for all the medications used. The results showed significant variability between patients in Cmax, Tmax, and AUC of tacrolimus, sirolimus, MPAG, and MPA. They stated that there are substantial differences in how patients with BS absorb immunosuppressant drugs. They indicated that there is a possibility that people undergoing a transplant and who have BS should need a higher immunosuppressant medicine dose than non-bariatric surgery patients, especially those who undergo bypass surgery [45].

## 4. Outcomes and Complications of Bariatric Surgery on Liver Transplantation

Endoscopic bariatric therapy has become a famous approach for individuals with obesity and NAFLD [46]. Devices in the gastric and duodenal system and techniques such as intragastric balloons, endoscopic-sleeve gastroplasty, small-bowel bypass, and duodenal-mucosal resurfacing via endoscopic procedures have been studied. Although the latter could interfere with the absorption of immunosuppressive drugs after LT, it can lead to stable body weight and be a long-term therapeutic approach [46,47]. Intragastric balloons have shown rapid weight loss, leading to improvements in NAFLD profile in the short term [48,49].

BS emerging procedures, such as laparoscopic greater curvature application, one anastomosis gastric bypass, and one anastomosis duodenal switch, still need more research on their impact on patients with liver diseases [50]. Considering that obesity has risen in the last decade, BS has become the therapeutic intervention for long-term weight loss without increasing comorbidities [51,52]. Moreover, in patients with ESLD and awaiting LT, obesity could worsen the prognosis [46]. Complications such as venous thromboembolism and thrombosis of the hepatic artery have been related to obesity complications in LT [53]. Even though complications have been well documented, several transplant organizations have still not set a specific BMI threshold and weight loss requirements for LT candidates [16,54]. Therefore, obesity represents a relevant challenge in surgical procedures, as it is linked to potentially high rates of complications.

Traditionally, obesity and MetS have been considered the leading risk factors for morbidity and mortality in surgical procedures. Consequently, LT surgeons are faced frequently with candidates for LT with morbid obesity, although obesity is considered a contraindication to LT [1]. Losing weight before LT remains a goal, and many non-surgical methods can be used to allow patients to reach the desired BMI and be finally listed for LT, which include physical activity, dietary patterns, behavioral therapy, and BS [16,51,55]. Although patients with obesity who are candidates for LT are requested to regulate their diet to lose weight before being listed for LT, there is no consensus on which assessment should be used to identify patients at risk of complications linked to obesity [16,51]. The categorization of obesity can be unprecise in individuals with ESLD due to intravascular volume status. It can be challenging to assess obesity risk due to weight fluctuation [55]. Fat deposition appears to predict morbidity and mortality in patients with cirrhosis strongly. Sarcopenic obesity, severe muscle depletion in obesity, is reported in 30–42% of these patients with cirrhosis [55].

As the popularity of BS has risen, so too have the complications associated with procedures including acute and chronic LF, and there is little data on liver deterioration after BS (Table 1). The first reported potentially life-threatening complications have been linked to jejunoileal bypass (JIB) [3,11]. Eilenberg et al. reported that 10 patients who developed liver dysfunction after BS presented liver alterations after a postoperative time of 15 months [56]. Liver dysfunctions were related to steatosis (70%), cirrhosis (30%), ascites (70%), hepatic encephalopathy (30%), and gastrointestinal bleeding (20%) [56].

LT and immunosuppression therapy effectiveness may be interfered with by BS. Sleeve gastrectomy (SG) has been related to better outcomes than other techniques such as Roux-en-Y gastric bypass (RYGB) [50,57]. The latter has shown concerns about potential malabsorption of immunosuppressive drugs, and inaccessibility of the gastric remnant after the procedure makes it a risky technique. Since SG is the simplest surgical technique, without digestive anastomosis, it reduces anastomosis-related complications [57].

The current surgical techniques are considered safe; however, there have been reported cases of liver function impairment after RYGB and one-anastomosis gastric bypass (OAGB). Deterioration of liver function occurred after RYGB (*n* = 5), OAGB (*n* = 1 (+1/conversion into OAGB)), and even gastric banding (*n* = 1) after 6 months postoperative. Symptoms included ascites (57%), hepatic encephalopathy (29%), and variceal bleeding (14%) [56]. For patients with compensated cirrhosis, BS has been proposed as part of the preparation for LT. Nevertheless, it may lead to severe postoperative complications that cirrhotic patients may not tolerate. This issue underlines the complexity of the treatment and creates the necessity for multidisciplinary care from specialists in both hepatology and bariatrics areas.

Although the application of BS has become an option in the last couple of years, the timing, the type of procedure, and the best identification candidates for this strategy are still under debate [58]. All the studies regarding BS report effective weight loss [55], between 21 and 75%, but with a high rate of complications ranging from 30 to 40%, and a mortality rate of 20% when gastric bypass has been performed [50].

Furthermore, the maintenance of digestive continuity preserves the functions of the pylorus, which assures gastric emptying and the absorption of essential nutrients such as the B vitamin complex, and minerals such as calcium and iron in the duodenum [57]. Additionally, SG has several complications that must be considered such as bleeding and splenic ischemia. However, the early postoperative mortality rate of BS has been estimated between 0.12 and 2.8%, and the most common complication is the staple line leak [57]. The prevention of this complication is still unpredictable, therefore, it requires a multidisciplinary team approach.

LT candidates are at high risk of sarcopenia and malnutrition, which may be worsened by interventions such as VLCD [16]. VLCD yields significant nutritional risks such as weight loss that may be accompanied by significant loss of FFM and/or sarcopenia [51]. Although the authors set the cutoff at 1000 kcal/day in patients with obesity and compensated cirrhosis, the quality of the diet in terms of macronutrient composition is important [16,51]. Data concerning the most appropriate preoperative diet in patients with morbid obesity scheduled for BS are still under discussion, despite several studies favoring preoperative weight loss [18,51]. Preoperative weight loss is not only intended for the benefit of the surgical procedure but also to work on the quality of a patient’s diet for adaptation during the postsurgical phase [2]. The preoperative period can also help to identify and correct vitamin, mineral, and trace-element deficiencies before surgery [59,60,61,62]. Furthermore, it improves IR, inflammation that results in decreased liver volume, and IFT, that ultimately facilitates the surgical procedure [63].

BS with advanced liver disease represents a challenge for transplant programs when decompensated, therefore, may be performed successfully in carefully selected patients with cirrhosis [50,57]. Although, weight loss by BS can facilitate transplant candidacy. If SG is performed in LT candidates, it can mean fewer concerns about impaired absorption of immunosuppressant drugs and nutrients after the transplant [50,57]. The largest study included 32 LT candidates with Child–Pugh class A and B cirrhosis who underwent laparoscopic SG and showed low complication rates compared to the general SG population [50,57,64].

Patients with decompensated cirrhosis and obesity are considered at greater risk of accessing BS before LT. This situation makes a simultaneous SG and LT attractive [50]. Patients who had SG at the time of LT had sustained weight loss 3 years after the transplant compared to those who had LT alone [50,57]. At three years after LT, patients treated with LT and SG maintained a significantly higher percentage of total body weight loss compared with the control group (34.8 ± 17.3% vs. 3.9 ± 13.3%; *p* < 0.001), with a lower prevalence of complication at follow-up [3,57].

BS after LT may not be a choice before transplantation or individuals tend to gain substantial weight after LT [50,65]. After any bariatric procedure, the number of calories that can be ingested is limited during the first 6 months after surgery when the weight loss is maximal and certainly below the threshold of 1000 kcal/day [16]. Consequently, the type of diet in this setting is very close to the VLCD model. Therefore, a 4-week course of omega 3 fatty acids during and after BS is advised to reduce inflammation [2,16,51]. Concerns have been raised that VLCDs produce significant nutritional risks, such as rapid weight loss accompanied by significant loss of FFM and/or sarcopenia [51]. Appropriate counseling before and after LT is necessary to prevent post-transplant obesity and its related complications [3]. There is no certainty that BS is a long-term therapeutic option for cirrhosis and BS, and further investigations are needed [65]. The impact of BS and long-term outcomes are well documented in the non-transplant population, however, more information from liver transplant individuals is still needed [66].

## 5. Future Perspectives

Obesity is considered a worldwide pandemic. Approximately 95% of patients develop NAFLD, sarcopenia, and myosteatosis. Some obesity-related liver complications are NAFLD, NASH, or hepatocellular carcinoma. Weight loss has a crucial positive impact on advanced chronic liver disease.

Regardless, other techniques used to lose weight before BS have become an option in recent years, though the type of surgical procedure and the correct classification of the candidates are still controversial. BS is considered a long-term corrective solution for morbid obesity, however, there are complications related to the procedure, including LF.

Candidates for LT with morbid obesity present more surgical risk, thus, the main objective is losing weight before the surgery. As a result, BS has been suggested to be part of the therapeutic plan for LT in patients with morbid obesity.

The changes in the anatomy of the gastrointestinal tract are another challenge for BS patients, and the absorption of nutrients and medication must be adjusted to the new morphophysiological situation; the plasma levels of micronutrients and the oral bioavailability of certain drugs need to be observed. The use of biomarkers is recommended mainly to avoid adverse effects. Some surgical procedures cause malabsorption of nutrients of 25% of protein and 72% of the fat consumed. Nutrient malabsorption may develop depending on the gut site affected by the surgery. It will also affect micronutrients; the reasons may be bypassing the duodenum and jejunum or the limited contact with the brush border or the gut. Vitamins D, A, E, nd K, and zinc are the main micronutrients with affected absorption. There is also a lack of iron, calcium, vitamin B12, and folate absorption. In addition, nutrient deficiencies can also occur because of inadequate nutrient dietary intake [60,61,62].

BS candidates who need an LT have a potential risk of taking fewer doses of medication as a result of the disturbances in the digestive anatomy. Blood drug levels can be up to 50% lower in BS patients, so the recommendation must be to prescribe higher doses to achieve serum levels. Furthermore, the use of immunosuppressants in the outcome of transplanted patients with morbid obesity is still under debate.

The perspectives of bariatric interventions in LT candidates represent clinical and surgical challenges due to the complexity of metabolic changes and the impact on the pathophysiology of these patients. There is a need for more studies to determine the best time to perform the BS as a potential benefit to decrease LT complications.

## 6. Cost-Effectiveness of Bariatric Surgery in Liver Transplantation

Patients with obesity face barriers in access to transplants and unique challenges in perioperative and postoperative care and its outcomes. These patients may not even be referred for transplant evaluation and are much less likely to be waitlisted or undergo a transplant [46].

The obesity prevalence is more common among patients transplanted for NASH than other indications. Additionally, it is a risk factor for liver disease and accelerates disease progression of other causes of cirrhosis [66]. It increases the risk of primary liver malignancies and increases BMI, which is a predictor of decompensation of liver cirrhosis [1]. The options for managing obesity in the transplant population are similar to the non-transplant population and include diet, exercise, and BS [46]. NAFLD progressing to NASH has arisen as the second leading indication for LT and has been related to the augmenting rates of hepatocellular carcinoma (HCC) with and without underlying cirrhosis [67]. BS can be effective but can also be considered an expensive treatment for patients with NASH [67].

BS has emerged as an effective weight-loss treatment and a potential therapy for NASH in individuals with obesity. The criteria for BS in the general population include careful screening of selected patients with a BMI of at least 40 or a BMI of 35 with one or more obesity-related comorbidities. Evaluating cirrhotic patients is not easy, but bariatric surgery can have further challenges, as some already have cognitive impairment due to hepatic encephalopathy [66].

It is known that BS is considered one of the most effective options to treat severe obesity and reduces obesity-related comorbidities, such as NAFLD. A 2017 publication mentioned that BS (bariatric procedures for laparoscopic RYGB) was cost-effective for NASH patients with obesity, disregarding the stage of fibrosis, and in overweight patients with advanced liver fibrosis. The analysis showed the potential value of BS and lifestyle changes for treating NASH [68]. Currently, there are no known guidelines on BS in patients with NAFLD or cirrhosis or a consensus on which bariatric modality is best for patients with this disease. Patients who undergo BS with hepatic diseases require close follow-up to continue even after the immediate postoperative period [66]. In a cost-effectiveness analysis in 2019 by Klebanoff et al. [69], they mentioned that BS could be highly cost-effective in patients with NASH in compensated cirrhosis and obesity or overweight patients. The findings from this analysis suggest that clinical trials can evaluate the effect of bariatric procedures in patients with NASH cirrhosis, including those with a lower BMI. In this simulation model study, laparoscopic SG had an incremental cost-effectiveness ratio of USD 66 119 per quality-adjusted life years in overweight patients [69].

Patients with obesity that undergo BS have demonstrated several benefits, such as an increase in life expectancy and quality-adjusted survival after surgery in patients in all classes of obesity—even those with a BMI between 30 and 35. Additionally, it has been stated that compared with lifestyle intervention, surgery leads to more quality-adjusted life years for all patients with obesity and overweight, regardless of fibrosis stage, and leads to superior outcomes in life expectancy for all cohorts of patients assessed [68].

As obesity is a rapidly growing worldwide epidemic, the relationship between LT and previous BS may be more common in the future. BS is even being used to favor access to LT in patients with obesity that would otherwise not be listed because their weight and BMI limit them from entering the transplant waiting list [70]

BS has proven to have economic benefits for the obesity burden. For example, Lauren et al. [71] found that BS’s effectiveness and cost-effectiveness vary depending on the baseline severity of type 2 diabetes (T2D). In the next five years, RYGB will be the preferred therapeutic strategy for patients with severe obesity (adults with severe obesity BMI ≥ 40) independently from the baseline T2D severity. RYGB continued as the most preferred bariatric strategy in most analyses, highlighting its effectiveness in managing severe obesity and T2D despite its higher costs and rates of surgical complications. It showed a cost-effectiveness threshold of USD 100.000 per quality-adjusted life years (QALY) gained [71].

All around the world, NAFLD is strongly associated with obesity and MetS with metabolic complications such as T2D. This rapid increase in NAFLD prevalence has significantly increased associated health-care and economic burdens [72]. A review of 976 Medicare beneficiaries with NAFLD who were required to be hospitalized from 1 January 2010 to 31 December 2010 had a median annual total payment of about USD 11,000, with significantly lower payment for patients without cirrhosis compared with those with cirrhosis (USD 10,146 vs. USD 18,804, *p* < 0.01) [73]. NAFLD is closely associated with MetS. It is common to find a simultaneous diagnosis of NAFLD in patients with existing T2D and is related to inadequate glycemic control with all T2D complications, increased risk of cardiovascular complications, and mortality [72,73]. A recent study from Iannelli et al. showed different data about the clinical and economic impact of the previous BS on LT. No significant differences were detected regarding hospitalization costs and re-hospitalizations between patients with a previous BS versus those with obesity without BS and undergoing LT [70].

Many complications are associated with BS and LT, including nutritional deficiencies. In most reports, LF occurred early postoperatively, presented with quick deterioration of liver function, and may have been associated with rapid weight loss and malnutrition. The effect of BS on liver function and the nutrition consequences in patients with cirrhosis, regardless of etiology, years after BS is not yet completely clear, but the association of increased intestinal permeability and bacterial overgrowth with severe protein malnutrition are common features that are found in this condition, generally associated with jejunoileal bypass [11].

Sarcopenic obesity, defined by the loss of muscle mass and preservation of fat, is a condition that can be present in patients with cirrhosis who have undergone BS, and this sarcopenia may have a role in the clinical outcomes of this group of patients with obesity. In 2019, Idriss et al. [74] mentioned that cirrhotic patients who underwent BS had increased rates of delisting and decreased rates of LT. The presence of malnutrition and sarcopenia may partly account for the worse outcomes in patients with a history of the previous BS on the LT waiting list [74]. Additionally, sarcopenic obesity causes a significant risk of physical impairment and disability that is higher than the risk induced by obesity and liver disease alone. Furthermore, it is an independent risk factor for chronic liver disease in patients with obesity, a negative prognostic marker for the evolution of liver cirrhosis, and it can affect the results of LT. Pre-transplant sarcopenia is widely recognized as associated with short-term survival after living donor liver transplantation [1]. The presence of malnutrition and sarcopenia among patients with BS may contribute to worse outcomes [74].

The main goal of performing BS after LT is to improve the survival rates by reducing obesity-related comorbidities and the incidence of recurrent NASH [66]. Before the transplant, the patients want to meet the list criteria. We must mention that the available epidemiological data on obesity in transplant candidates and recipients fail to account for patients who are too obese to be considered for transplant listing. Most transplant centers endorse using BMI cutoffs for transplant listing. However, significant weight loss with lifestyle interventions may not always be possible for all patients with obesity, especially with end-stage organ disease [75]. Additionally, undertaking BS prior to LT can have major consequences such as delaying the receipt of the organ while the patient awaits adequate BMI response and addresses potential complications from BS [66].

A plan for LT candidates with obesity is to lose weight and lower BMI so that patients may meet the correct weight-listing requirements and reduce the risk of perioperative transplant morbidity and mortality. BS performed simultaneously with LT may be linked to high preoperative risks. It has been described that the ideal time to perform BS in patients with cirrhosis is before portal hypertension develops. Even though there is no consensus or clear guideline established on the specific Child–Pugh score threshold for candidates for BS, the goal of compensated cirrhosis is the most desirable, or at least a year after LT to minimize the risk of rejection from interruptions in immunosuppressant therapy [66]. BS and LT concurrently performed can provide some benefits such as decreased costs, diminished hospital stay, and reduced pain and stress. A study by Zamora-Valdes et al. concluded that combined LT and SG resulted in more effective and durable weight loss and fewer metabolic complications at last follow-up [76] (Table 2).

BS for patients with obesity has developed a potential public health role in the rising number of LT performed for NASH cirrhosis. Currently, there are no guidelines on BS in patients with cirrhosis or a consensus on which bariatric modality is best for these patients. There is a need for more data and information for the correct and optimal approach for LT for patients with obesity [68].

## 7. Conclusions

NAFLD and NASH are common obesity-related liver complications. Additionally, obesity is an expected condition in patients with NAFLD after LT. It can also affect graft dysfunction and predispose them to an altered nutritional status. Hence, these patients could benefit from BS before LT.

It is important to remember that body composition can be essential for cirrhotic patients, mainly due to fat distribution and specific conditions such as malnutrition and sarcopenia. It can be used to predict the outcome of LT. Nevertheless, even though losing weight is fundamental for better outcomes in LT, it is crucial to consider the type of diet these patients will have to avoid other complications. For example, VLCDs can worsen the graft’s clinical course. The energy and calorie restriction diet helps control IR, hepatic insulin sensitivity, glucose tolerance, and liver fibrosis.

Patients undergoing BS and LT suffering from NAFLD-related cirrhosis can benefit from the weight loss of the procedure. Bariatric interventions in LT candidates/recipients would positively change their metabolism and liver pathophysiology. Nevertheless, there are still controversial positions about the cost-effectiveness of the best BS procedure and the time to perform it for LT patients.

BS is a long-term corrective solution for morbid obesity; however, there are complications related to the procedure, including LF. Other bariatric procedures for this condition, such as intragastric balloons and gastric bypass, have greater long-term complications. BS has been suggested to be part of the therapeutic plan for LT in patients with morbid obesity. Obesity in ESLD patients awaiting LT could worsen prognosis due to surgical and cardiovascular complications.

When BS is carried out on a patient with a liver condition it hardly worsens the liver function or the potential need for an LT. Nonetheless, a few cases of LF after BS have been reported, which requires LT. Despite its low frequency, these patients can develop malnutrition and bacterial overgrowth. Because of all these considerations, candidates with extrahepatic comorbidities must carefully analyze the surgical option, particularly those restrictive procedures.

Additionally, the impact of BS on the absorption and effectiveness of immunosuppressant drugs must be considered, especially in RYGB procedures. It is recommended for the candidates undergoing LT with BS to receive higher immunosuppressant medication than non-bariatric patients.

The authors recommend BS before the LT due to the better outcomes for patients who lose weight before the procedure, as presented in this review. Furthermore, it is essential to have a multicentric study to evaluate immediate and long-term outcomes in candidates/recipients undergoing LT since there are several results in these patients with obesity and NASH and an increased rate of surgically related complications for LT recipients.

## Figures and Tables

**Table 1:** Pre- and post-LT complications [4].

Common Complications with NAFLD before Liver Transplantation	Common Complications with NAFLD after Liver Transplantation
Diabetes mellitus Cardiovascular disease Kidney dysfunction Altered nutritional status	Diabetes mellitus Obesity Cardiovascular disease Kidney dysfunction Liver failure Graft dysfunction Altered nutritional status

**Table 2:** Cost-effectiveness of bariatric surgery in liver transplantation.

Cost Effectiveness of Bariatric Surgery in Liver Transplantation		
Before Liver Transplantation	At Liver Transplantation	After Liver Transplantation
Meet the right weight-listing requirements Reduce the risk of perioperative transplant morbidity and mortality	Fewer metabolic complications at follow-up More effective and durable weight loss	Improve survival rates Reduce obesity comorbidities Reduce after transplant-related obesity

## Data Availability

Not applicable.

## Abbreviations

BS—bariatric surgery; NAFLD—non-alcoholic fatty liver disease; NASH—non-alcoholic steatohepatitis; IL—interleukin; TNFa—tumour necrosis factor-alpha.
